# Nutritional Value Evaluation of New Pea Genotypes (*Pisum sativum* L.) Based on Their Chemical, Amino Acids and Dietary Fiber Composition

**DOI:** 10.3390/molecules29215033

**Published:** 2024-10-25

**Authors:** Anna Fraś, Marlena Gzowska, Magdalena Wiśniewska

**Affiliations:** Plant Breeding and Acclimatization Institute—National Research Institute, Radzików, 05-870 Błonie, Poland; a.fras@ihar.edu.pl (A.F.); m.gzowska@ihar.edu.pl (M.G.)

**Keywords:** amino acids, pea, protein, starch, dietary fiber

## Abstract

This research concerned the assessment of the utility value of new pea breeding materials intended for registration in the European Union. The research material consisted of sixteen breeding lines and four reference pea varieties. The evaluation was carried out based on the chemical composition of seeds and its variability within the studied genotypes. The contents of protein, starch, lipids, ash and dietary fiber (DF) were determined. The nutritional value of the protein was assessed in vitro using the value of the limiting amino acid index chemical score (CS) and the integrated essential amino acid index (EAAI). The analyzed pea genotypes were characterized by significant diversity in terms of the content of the tested components. The obtained results combined with the PCA analysis were used to select pea genotypes of the highest quality, having the potential as a raw material for the production of food with health-promoting properties. The effect of the conducted research was the identification of three pea genotypes with the greatest potential in terms of protein content and dietary fiber complex.

## 1. Introduction

It is estimated that by 2050 the world population will reach 9.6 billion people [1]. As a result, an additional 260 million tons of protein will be needed to meet the needs of the world population; therefore, it is necessary to look for alternative food sources, including those of plant origin, to ensure food security. Legume-based food plays an important role in this process, since it is a rich source of calories, protein and other valuable ingredients. Legumes, in addition to cereals, play an important role in human nutrition, among which the most popular are beans, peas, broad beans, chickpeas, lentils and soybeans. In developed countries, this type of product is increasingly used as a part of a healthy diet. Besides legumes in raw form, the most popular legume products include tofu, tempeh, plant drinks, flours and functional ingredients of bakery products, pasta, protein supplements, protein granules as well as vegan food products such as ready-to eat foods like burgers, sausages or purees.

In recent years, peas, the fourth most cultivated legume crop worldwide, grown for human nutrition as well as livestock feeding, have become increasingly popular in this area [2,3,4]. Pea (*Pisum sativum* L.) belongs to the Leguminosae family, which includes 800 genera and 20,000 species of plants. Pea is a diploid plant (2n = 14), and is annual, with a short vegetation period. To grow and develop, it needs a cooler climate with moderate air and soil humidity, which guarantee good yields. Peas, like other legumes, play an important role in modern agriculture. This is due to their ability to establish symbiosis with nodule bacteria and absorb nitrogen from the atmosphere. In this way, they increase the level of nitrogen in the soil and reduce the use of nitrogen fertilizers and greenhouse gas emissions into the atmosphere. In the case of peas, their carbon footprint is one of the smallest among legumes [5,6]. Due to its adaptability and good nutritional and economic value, this crop is widely grown worldwide, especially in Europe, North America, Asia and Australia [4]. Another reason to promote plant-based food sources is the climate crisis. Almost 14.5% of global greenhouse gas emissions are attributed to animal production. Therefore, increasing the production of alternative protein sources will have a measurable positive impact on the environment.

The beneficial effects of the pea on human health can be attributed to its chemical composition. The pea is characterized by quite a low level of lipids and a high content of protein, carbohydrates, minerals, dietary fiber and phenolics. Protein is the most important nutrient in pea seeds and constitutes 15–30% of the dry matter. Due to the complexity of protein genes encoding peas and environmental factors, the protein composition of peas can be different depending on the source of the pea [2,5]. The protein fraction in pea seeds consists mainly of globulins and albumin storage proteins. The main storage proteins are the globulins legumin and vicilin. Vicilin proteins contain lower amounts of the sulfur-containing amino acids methionine and cysteine than legumin. These have been identified as limiting amino acids in peas. On the other hand, pea seeds are relatively rich in lysine—therefore, their essential amino acids profile is complementary to that of cereal grains [5,7,8,9]. Due to its quality, pea protein, together with soy, wheat and rice proteins, is used in meat analogues [10]. Moreover, pea protein is used in food and pharmaceutical products, including dietary supplements, fortified drinks, and protein mixtures [11]. Starch is the second most important nutrient component in pea seeds and contributes about 40–50% of the dry weight. The main advantages of pea starch are its high amylose level, resistance to shear thinning, rapid retrogradation and high content of resistant starch. Research on the structure and functionality of pea starch has been conducted for years, and its full characterization is important for food processing to improve the texture of pea-based products like frozen foods, cookies, sauces or soups, while also improving consumer acceptance [12,13]. Another important complex of health-promoting ingredients in pea seeds is dietary fiber, ranging from 9.0% to 32.6% of dry weight [14,15,16,17]. Pea seed fiber consists mainly of the insoluble fraction of non-starch polysaccharides, followed by raffinose family oligosaccharides, as well as resistant starch, lignin and related substances, and uronic acids.

The well-balanced chemical composition of pea seeds makes this plant a potentially ideal raw material from a nutritional point of view, providing all the necessary nutrients. The health benefits of consuming legumes, including peas, have been widely described in the literature. These include, among others, improved glycemic and insulinemic control, the prevention of type 2 diabetes and a lower risk of cardiovascular diseases, hypercholesterolemia, and hypertension [14,18,19].

Currently, intensive breeding works are being carried out all over the world, which concern not only the size and stability of the yield, but also its resistance to biotic and abiotic stresses. An important aspect of this work is improving the chemical composition of seeds. The work is aimed at increasing the protein content, while maintaining its quality, and also improving the content of starch, especially resistant starch, which affects the level of glucose in the blood. Thanks to the discovered pea mutants, it is possible to carry out work aimed at reducing the content of anti-nutritional ingredients such as inhibitors and phytates [5]. Considering the growing demand for high-protein plants and the development direction/trend in pea breeding and use in recent years, it is important to look for genotypes and varieties that, in addition to high yield, will be distinguished by the highest possible content of nutritional and health-promoting molecules. Therefore, the aim of the presented research was to analyze the content of nutrients and dietary fiber, and assess the variability of these compounds in the newest pea breeding lines intended for registration in the European Union in relation to the varieties available on the market.

## 2. Results and Discussion

### 2.1. Nutrient and Amino Acid Composition

The growing importance of peas is mainly due to their high nutritional value. The content of nutrient components in the analyzed pea genotypes and the results of variance analysis are presented in Table 1. For comparison purposes, the results of four pea varieties are also included. The analyzed pea breeding materials differed significantly in terms of protein content, which was on an average level of 246.6 g kg^−1^, ranging from 211.0 g kg^−1^ for the WTD5005 line to 301.9 g kg^−1^ for the WTD5006 line. These values were slightly higher compared to the described pea varieties, for which the average protein content was 237.3 g kg^−1^, ranging from 197.8 g kg^−1^ for the Cysterski variety to 268.5 g kg^−1^ for the Starski variety. Among the analyzed pea materials, 12 breeding lines were distinguished by protein content above the average obtained for the varieties, which is a good prognosis for their future registration as high-protein varieties. Tobiasz-Salach et al. [20] described the protein content in the pea varieties analyzed over three consecutive harvest years at an average level of 218.3 g kg^−1^, ranging from 188.0 g kg^−1^ to 235.0 g kg^−1^, which is a slightly lower value in comparison to the presented results. A lower protein content, at an average level of 208.8 g kg^−1^, was also determined by Woźniak et al. [21] for pea varieties, harvested in three different tillage systems. Higher protein values, between 260 g kg^−1^ and 320 g kg^−1^, were observed by Vidal-Valverde et al. [22], who also analyzed pea breeding lines, while the most comparable values at an average level of 263.6 g kg^−1^ were obtained by Costantini et al. [3]. The significantly higher protein content in the tested breeding lines in comparison to the varieties and most of the literature data confirms their high nutritional value. The various protein contents in pea seeds presented in the literature depend, among other things, on the influence of genotype, environment, type of agrotechnics used and sowing density.

With regard to other nutrients contained in pea seeds, significant, although small, differences were found in the contents of lipids and ash. The average lipids content in the lines and varieties was at a level of 30.2 g kg^−1^, ranging from 26.6 g kg^−1^ for the HR-G6 line to 36.9 g kg^−1^ for the WTD5006 line, while the ash content ranged from 26.4 g kg^−1^ for the WTD5005 line to 35.6 g kg^−1^ for the WTD5006 line, with an average content at the level of 30.9 g kg^−1^. Pea lipids, despite their low content, have significant health benefits and are a source of monounsaturated fatty acids. A similarly significant differentiation within genotypes, but with a higher content of determined ash and lower amount of lipids, has been obtained by other researchers [23,24]. In their study, the level of ash found was in the range between 30.0 g kg^−1^ and 39.3 g kg^−1^, whereas the average lipids content was 23.7 g kg^−1^. Chen et al. [2] found a lipids content in pea varieties in the range of 5.7 g kg^−1^ to 35.2 g kg^−1^ and obtained an average ash content similar to the results described above, at the level of 28.2 g kg^−1^. Different results with regard to lipids content were obtained by Bähr et al. [16], who found the amounts of these compounds were at an average level of 60 g kg^−1^ for the three pea genotypes tested. The obtained differences in the contents of the above components may depend on both the pea genotype itself and environmental conditions, including the plant cultivation system and the analytical method used [21,25].

Besides the protein, starch plays an important nutritional role in pea seeds, because it is a source of carbohydrates. Among the legumes, peas are one of the richest sources of starch, and as a consequence also resistant starch, which is a part of the insoluble fraction of dietary fiber. Its content, like other nutrients, varied significantly in the tested genotypes. The amount of starch in the breeding lines ranged from 268.9 g kg^−1^ for the WTD5006 line to 441.4 g kg^−1^ for the WTD5008 line, at an average level of 406.2 g kg^−1^. The WTD5006 breeding line was the genotype with the lowest amount of starch and the highest content of other nutrients like protein, lipids and ash. The analyzed pea varieties were characterized by a higher starch content compared to the breeding lines, at the level of 448.9 g kg^−1^, ranging from 419.7 g kg^−1^ for the Olimp variety to 466.1 g kg^−1^ for the Akord variety. Starch content was negatively correlated with protein content (r = −0.71, *p* < 0.01), since these are the major constituents in inverse proportions in the bulk of the pea. The most similar range of starch content compared to the tested lines was determined by Woźniak et al. [21], who obtained values between 249.8 g kg^−1^ and 431.2 g kg^−1^ in various years and cultivation systems. A similar average value for the tested varieties, at the level of 428.2 g kg^−1^, was described by Zhang et al. [15], while Nikolopoulou et al. [26] observed amounts of starch in pea varieties ranging from 334.0 g kg^−1^ to 475.0 g kg^−1^ during two years of research.

The nutritional value of protein depends primarily on the content of amino acids, in particular essential amino acids (EAAs), and their mutual proportions [27]. These amino acids, also called exogenous, cannot be synthesized in human and animal cells or are synthesized in insufficient quantities, and must be supplied in food. Their absence or limited quantity reduces the biological value of the protein and prevents its use. The group of EAAs includes histidine (His), isoleucine (Ile), leucine (Leu), lysine (Lys), methionine (Met), phenylalanine (Phe), threonine (Thr), tryptophan (Try) and valine (Val) [28,29,30]. Pea protein is considered a high-quality protein, which meets FAO and WHO recommendations regarding the availability of amino acids in appropriate proportions, as well as the availability of EAAs, except for methionine [31]. To expand the protein quality analysis, the sum of amino acids (AA) and the EAA composition of pea genotypes were analyzed, and the obtained results are presented in Table 2. When assessing pea protein, the amount of semi-essential amino acids—cysteine (Cys) and tyrosine (Tyr)—was taken into account. The amino acid profile of the tested genotypes was characterized by significant differences in both of these parameters. The average content of the AA for the tested material was 94.6 g/100 g of protein, and ranged from 92.9 g/100 g of protein for the protein of the HR-G8 pea breeding line to 96.7 g/100 g and 96.8 g/100 g of protein for the protein of the WTD5001 breeding line and the Akord variety, respectively. Approximately 40% of the sum of all amino acids were essential amino acids at the average level of 37.8 g/100 g of protein. The literature presents similar contents of EAA determined in pea protein in the range of 35.9–44.4 g/100 g of protein, the share of which in the total amino acids was on average 30.8% to 39.3% [22,27,32]. The authors of these studies showed that seeds with an uneven, wrinkled surface contain approximately 0.36% more EAA compared to seeds with a smooth surface [32]. In the presented work, more than half of the tested pea genotypes were characterized by a higher EAA content in comparison to the average obtained for the entire material. The lowest amounts of EAA were in the WTD5003 and WTD5004 lines, at the level of 35.5 g/100 g and 36.2 g/100 g of protein, respectively. The protein of the HR-G8, HR-G7 and HR–G3 genotypes contained the most EAA, at the levels of 39.0 g/100 g, 39.1 g/100 g and 39.2 g/100 g of protein, respectively. Among EAAs, the highest amounts were Lys (7.9 g/100 g of protein) and Leu (7.1 g/100 g of protein). The content of the first of the mentioned amino acids was in the range of 7.1–8.4 g/100 g of protein in the WTD5003 and HR-G 3 pea proteins, respectively.

In the protein of crop plants such as cereals, Lys is a deficient amino acid. In the case of peas, sulfur-containing amino acids are deficient. The sum of methionine and cystine in the presented study amounted to an average of 2.6 g/100 g of protein, whereas extreme amounts were determined in the pea protein of the WTD5003 (2.3 g/100 g of protein) and WTD5005 (2.8 g/100 g of protein) breeding lines. Similar contents of lysine and the sum of sulfur amino acids at the level of 7.7 g/100 g of protein and 2.6 g/100 g of protein, respectively, were shown by Wiśniewska et al. [27]. A slightly lower level of Lys content (7.1–7.4 g/100 g of protein) and Met (1.06–1.1 g/100 g of protein) was obtained by Shelepin et al. [32]. A significant part of the AAs were non-essential amino acids (NEAA) (endogenous) that can be synthesized de novo in adequate amounts by the animal organism to meet requirements for growth, development, and health, and therefore need not be provided in the diet [28,29,30].

In the presented study, the content of individual NEAA varied significantly (Table 3). The amount of NEAA was determined at an average level of 56.8 g/100 g of protein, and quantitatively, these contents were mainly represented by aspartic acid (Asp) and glutamic acid (Glu), at the average level of 11.9 g/100 g and 17.3 g/100 g of protein, respectively. These amounts were on average 9.8% lower than those presented by Shelepin et al. [32]. The proteins of the tested pea genotypes also contained significant amounts of arginine (Arg), ranging from 7.3 g/100 g to 10.1 g/100 g of protein. The remaining endogenous amino acids were present in much smaller amounts, each less than 5 g/100 g of protein. The obtained contents of individual NEAAs correspond to the data in the literature [32]. The nutritional value of the protein was assessed based on the value of the limiting amino acid index chemical score (CS) and the integrated essential amino acid index (EAAI). The chemical assessment of amino acids (CS) involves comparing the amino acid composition of the tested protein with the composition of the reference protein, which is chicken egg [33]. In turn, EAAI is the geometric mean of all CS values [34]. The results are presented in Table 2. The study shows that the protein of the tested pea genotypes was well balanced in terms of most amino acids. In the case of the tested pea genotypes, the limiting amino acid was the sum of methionine and cysteine, for which the average CS value was 46%. The sum of sulfur amino acids varied significantly but slightly, and ranged from 2.3 g/100 g protein for the WTD5003 line to 2.8 g/100 g of protein for the WTD5005 line. The average value of EAAI obtained for the tested pea genotypes was 82, ranging from 76 for the WTD5003 breeding line to 85 for HR-G 3. The seeds of the HR-G3 breeding line had the best protein nutritional value, as indicated by the value of the limiting amino acid index (CS = 49) and the integrated exogenous amino acid index (EAAI = 85). Pea seeds of the lines WTD5005 (CS = 49; EAAI = 83), WTD5008 (CS = 48; EAAI = 84) and HR-G8 (CS = 48; EAAI=84) were also characterized by high protein nutritional value, assessed on the basis of CS and EAAI indices. The value of the EAAI index was significantly correlated with the sum of EAA (r = 0.98, *p* < 0.01) and with the amounts of lysine (r = 0.80, *p* < 0.01), isoleucine (r = 0.79, *p* < 0.01), valine (r = 0.77, *p* < 0.01) and threonine (r = 0.72, *p* < 0.01), as well as the sum of sulfur amino acids (r = 0.65, *p* < 0.01).

### 2.2. Dietary Fiber and Its Components

In addition to a large amount of protein and other nutrients, peas are also an important source of dietary fiber with a very beneficial composition. This makes this plant one of the best plant foods for humans. The content of dietary fiber (DF) and the amounts of its individual components in the analyzed material are presented in Table 4. The contents of DF varied significantly, and the values ranged from 217.6 g kg^−1^ to 362.4 g kg^−1^ for the WTD5007 and HR-G2 lines, respectively. Smaller differences were observed in other varieties, in the range between 229.0 g kg^−1^ for the Olimp variety and 288.4 g kg^−1^ for the Akord variety. The high DF content in pea seeds makes their health value very high. Nowadays, DF consumption in the population is much lower than the recommended level. According to the EFSA (European Food Safety Authority) the adequate intake of DF for adults is 25 g per day. With regard to the above data, the consumption of 100 g of pea covers this demand almost 100%. The content of DF in peas described in the available literature varies greatly, which is related to the different methods of its determination. There are no data regarding the results of DF content obtained using the method described in the manuscript, but there are many references regarding individual fiber components. Similar, although slightly lower, fiber contents were described by Zhang et al. [15], who presented an average amount of 229.8 g kg^−1^, while Kumari and Deka [35] described a range of fiber content from 140.0 g kg^−1^ to 260.0 g kg^−1^, including the insoluble fraction (IDF) in the range between 100.0 g kg^−1^ and 150.0 g kg^−1^ and the soluble fraction (SDF) ranging from 20.0 g kg^−1^ to 90.0 g kg^−1^. Bähr et al. [16] also determined a fiber content in pea varieties from 91.0 g kg^−1^ to 189.0 g kg^−1^, which constituted lower values compared to those described in this publication.

The same authors also described the fiber components, for which they obtained values in the following ranges: SDF from 15.3 g kg^−1^ to 117.9 g kg^−1^, IDF from 71.4 g kg^−1^ to 112.6 g kg^−1^ and hemicellulose from 51.9 g kg^−1^ to 90.4 g kg^−1^, these results also being different compared to those described above. The content of DF was also determined by other authors [23,36] who described comparable, although slightly lower, results than those obtained in this study, ranging from 220.3 g kg^−1^ to 307.2 g kg^−1^. The contents of IDF and SDF fractions obtained by these researchers were in the ranges of 193.2 g kg^−1^ to 231.0 g kg^−1^ and 17.3 g kg^−1^ to 80.1 g kg^−1^, respectively. The main components of DF are non-starch polysaccharides (NSP), which constituted on average 51% of its total content in the tested material. Among these polysaccharides, the vast majority was the insoluble fraction (I-NSP), which constituted on average 93% of NSP. Its content was the most diverse among the breeding lines, for which values ranged from 87.2 g kg^−1^ for the WTD5003 line to 218.2 g kg^−1^ for the HR-G2 line. Much smaller differences in I-NSP, in the range between 113.9 g kg^−1^ and 144.0 g kg^−1^, were found in the case of the tested Olimp and Cysterski reference varieties, respectively. Significant differences, although small, were observed for the S-NSP fraction. Pea seeds, compared to other plant products including cereals, contain a high content of the S-NSP fraction, which has an important physiological impact. The SDF fraction is responsible for slowing down the passage of food in the digestive tract as well as the feeling of satiety. The values for both varieties and breeding lines were at a comparable level, ranging from 7.5 g kg^−1^ for the HR-G1 genotype to 10.9 g kg^−1^ for the Akord variety. In the case of NSP content, Nikopoulou et al. [26] and Gdala and Buraczewska [37] reported similar results to those presented above, and the obtained values ranged from 120.0 g kg^−1^ to 201.4 g kg^−1^. In turn, Wiśniewska et al. [27] determined an NSP content in pea seeds at the level of 120.0 g kg^−1^, of which the insoluble fraction was over 88%. The second most abundant fiber component after NSP was oligosaccharides (RFO—rafinose family oligosaccharides). These compounds do not undergo hydrolysis in the human digestive tract. When consumed in large quantities, they cause flatulence, diarrhoea and abdominal pain. In small amounts (3 g per day), they provide food for the microflora of the large intestine and stimulate its growth [38]. The average amount of RFO in the tested material was at a level of 79.5 g kg^−1^. Among legumes, pea seeds contain one of the highest levels of these substances, from 23.0 g kg^−1^ to 96.0 g kg^−1^ of dry matter [39]. The lowest values of 69.2 g kg^−1^ and 69.8 g kg^−1^ were found for the HR-G7 line and the Olimp variety, respectively, while the highest RFO contents, at levels of 88.3 g kg^−1^, 87.4 g kg^−1^, 87.2 g kg^−1^ and 86.7 g kg^−1^, were recorded for the WTD5001, WTD5004, WTD5003 lines and the Akord variety. Other authors [26] examining the RFO content in pea varieties grown in two consecutive years obtained similar values, ranging from 57.7 g kg^−1^ to 72.8 g kg^−1^. A wide range of RFO content in pea breeding lines, from 56.5 g kg^−1^ to 99.1 g kg^−1^, was also determined by Gawłowska et al. [39], whose results are similar to those obtained in our research. The other components of DF occurring in smaller amounts were uronic acids (UA), resistant starch (RS) and lignin. The analyzed pea genotypes differed significantly in terms of the content of these substances. The average content of UA in the tested breeding lines was higher compared to the varieties, and the values were at a level of 25.5 g kg^−1^ and 20.6 g kg^−1^, respectively. Extreme values for the tested pea lines ranged from 21.5 g kg^−1^ for the WTD5007 line to 33.9 g kg^−1^ for the HR-G8 sample. The values ranged from 18.4 g kg^−1^ to 23.0 g kg^−1^ for Olimp and Cysterski samples, respectively.

The content of RS in the analyzed pea lines ranged from 10.3 g kg^−1^ to 41.5 g kg^−1^ for the WTD5003 and WTD5006 genotypes, respectively, and from 14.6 g kg^−1^ to 30.7 g kg^−1^ for the Olimp and Akord varieties, respectively. The lignin content in the tested pea lines was the most diverse parameter among all the analyzed features, and the obtained values ranged from 2.7 g kg^−1^ for the WTD5007 line to 30.2 g kg^−1^ for the WTD5005 line. In the case of the pea varieties tested, the variation was small and the lignin value was relatively low, at an average level of 4.5 g kg^−1^. The observed variability of the Klason lignin content in the tested material is probably due to the research method used. During the test, the contents of lignin and accompanying compounds, such as waxes, cutins, suberins, saponins, phytates and tannins, were determined, all of which influence the final content of Klason lignin in the seeds. Other authors also examined the contents of the three ingredients described above. Wiśniewskas’ team [27] and Gdala and Buraczewska [37] obtained a UA content comparable to that in our research, ranging from 21.1 g kg^−1^ to 26.0 g kg^−1^, and a lignin content ranging from 2.0 g kg^−1^ to 30.0 g kg^−1^. Wiśniewska et al. [27] also determined the amount of RS at the average level 26.0 g kg^−1^. Similar RS contents were described in studies by De Almeida Costa et al. [23], who determined the level of this ingredient at 24.5 g kg^−1^, while pea seeds tested by Goodarzi Boroojeni et al. [40] contained 32.5 g kg^−1^ of RS. In turn, Kan et al. [36] presented a wider range of the content of RS in commercially available pea seeds, from 18.4 g kg^−1^ to 69.5 g kg^−1^. The reason for such notable differences in the contents of DF and its components may be the research material itself, which consisted of breeding lines with varying degrees of breeding advancement. Such material, when selected in the breeding process, is usually characterized by greater variability compared to stabilized varieties. Apart from the genotype (G), the environmental factor (E) and the interaction of G × E play an important role in shaping the chemical profile of pea seeds [26,41].

When analyzing the results obtained for the contents of nutrients and DF in the seeds of the tested peas, the chemical profiles of several of them are noteworthy. The WTD5006 breeding line was characterized by the highest contents of protein, lipids, ash and RS, and one of the highest amounts of UA and RFO, and consequently also DF. Breeding lines HR-G1, HR-G2, HR-G3, HR-G4 and HR-G8 were characterized by a slightly lower protein level in seeds. The nutritional values of the proteins of two of the mentioned genotypes, HR-G3 and HR-G8, assessed on the basis of CS and EAAI indices, were the highest, which was probably due to the highest content of the sum of EAA and the sum of methionine and cystine. The seeds of the HR-G2, HR-G3 and HR-G4 breeding lines also had the highest amounts of DF, NSP and I-NSP. HR-G1 and HR-G8 pea seeds also contained significant amounts of DF. In turn, the WTD5007 and WTD5003 breeding lines had the lowest DF content in the dry matter of seeds. The second of the mentioned genotypes also contained the protein with the lowest nutritional value. Analyzing the chemical profile of the seeds of the reference varieties, it can be concluded that they differed significantly, but only slightly. In terms of protein content, the varieties can be classified into two groups, of which seeds of the Akord and Cysterski genotypes had a lower amount of protein. However, the CS and EAAI indices were very similar for all four varieties. With regard to the DF content, a similar situation was observed—Akord and Cysterski contained more DF, including NSP and I-NSP, than the other two genotypes. In particular, the Olimp variety contained smaller amounts of DF and its individual components, RFO, UA and RS.

### 2.3. Principal Components Analysis (PCA)

**Figure 1:**
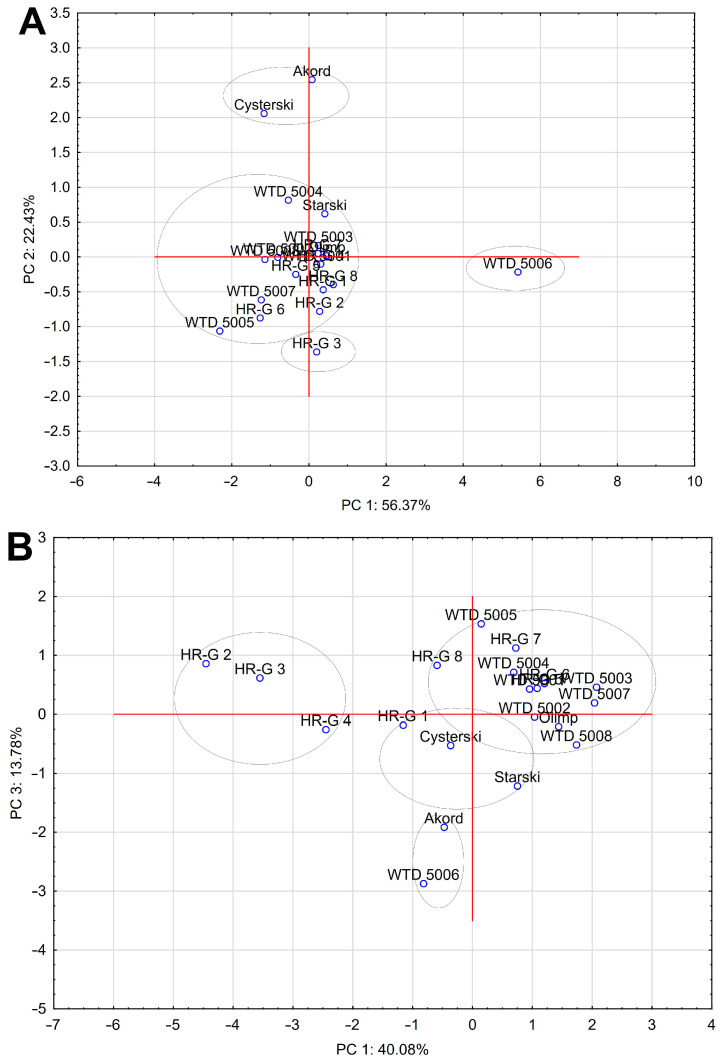

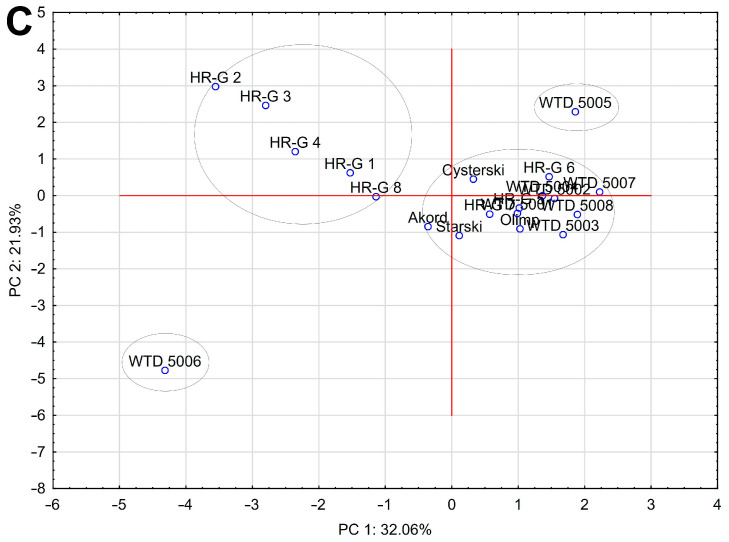
Score plot for principal components from analysis of pea genotypes: (**A**) nutrients; (**B**) dietary fiber; (**C**) all traits together.

To illustrate the differences between individual varieties, PCA analysis was used, separately for nutrients and dietary fiber components and for all traits together. The results of PCA analysis are presented in Figure 1. In the case of nutrients (Figure 1A), the two principal components, PC1 and PC2, explained 56.37% and 22.43% of the variation, respectively. The PC1 component was dependent on the protein (r = 0.82), starch (r = −0.83), lipids (r = 0.70) and ash (r = 0.64) contents, whereas the PC2 component was dependent only on ash content (r = 0.60). Four groups were chosen among the analyzed pea genotypes. The first group consisted of Akord and Cysterski varieties, the second group included the WTD5006 breeding line with positive PC1 value and negative PC2, whereas the third group included the HRG-3 breeding line. The remaining genotypes constituted the fourth, and largest, group. The groups created confirm the above-described results. The Akord and Cystreski varieties featured the highest levels of starch and lipids and the lowest amount of protein, whereas line WTD5006 contained the highest level of protein, ash and lipids and the lowest level of starch. In the case of DF and its components (Figure 1B), the three principal components, PC1, PC2 and PC3, accounted for 71.86% of total variability. The PC1 component had an inversely proportional impact on the I-NSP (r = −0.96), NSP (r = −0.97) and DF (r = −0.99) content. The PC2 component also had an inversely proportional impact on the RFO (r = −0.79) and S-NSP (r = −0.62) contents, whereas the PC3 component was dependent on RS (−0.86) and lignin (r = 0.53) contents. The described relationships are best illustrated by the graph between the PC1 and PC3 components, in which four groups have been selected. The first group consists of HR-G2, HR-G3 and HR-G4 genotypes with the highest level of DF and I-NSP fraction. The second group, according to a previous description, includes the Akord variety and WTD5006 breeding line, characterized by the highest contents of RS among all analyzed genotypes. The third group consisted of the line HR-G1 and the Cysterski and Starski varieties, whereas the biggest fourth group included other pea genotypes. When all traits were analyzed together, the variation between genotypes was explained by the four principal components PC1, PC2, PC3 and PC4, which accounted for 32.06%, 21.93%, 14.03% and 11.64% of the variation, respectively. The relationships between the PC1 and PC2 components are presented on a graph, and account for the largest share in the variability (Figure 1C). The PC1 component was dependent on the contents of protein (r = −0.69), starch (r = 0.63), ash (r = −0.56), RS (r = −0.54), I-NSP (r = −0.82), NSP (r = −0.82) and DF (r = −0.87), while in the case of PC2, it was the concentrations of lipids (r = −0.77), ash (r = −0.50), RS (r = −0.54), I-NSP (r = 0.51), NSP (r = 0.52) and lignin (r = 0.61) that were important. For the PC3 component, an inversely proportional impact was obtained for the contents of RFO (r = −0.66) and S-NSP (r = −0.66), and the PC4 component was dependent on starch (r = −0.52), ash (r = −0.51) and UA (r = 0.63) contents. Four groups were chosen among the analyzed genotypes. The first group consisted of the WTD5006 breeding line with negative values of both components and the highest level of RS. The second group included the genotypes HR-G1, HR-G2, HR-G3, HR-G4 and HR-G8 with negative PC1 values and positive or close to zero values of PC2. These lines also showed the highest levels of I-NSP among all analyzed genotypes. The third group included the WTD5005 breeding line with both positive values and the largest amount of lignin, while the last group consisted of 13 other genotypes of pea.

## 3. Materials and Methods

### 3.1. Materials

The material comprised sixteen Polish breeding lines of pea (*Sativum* L.) supplied by the Plant Breeding Strzelce Ltd. Co. (Strzelce, Poland) (eight genotypes named WTD5001-WTD5008) and Poznańska Hodowla Roślin Ltd. (Poznań, Poland)—Seed (eight genotypes named HR-G1—HR-G8), and were grown in one locality and harvested in 2021. For comparative purposes, four pea varieties (Akord, Cysterski, Olimp, Starski) grown in the same location were included in the study. The material for the study was selected by the breeders based on agronomic parameters, including yield. Peas were harvested in pods, and then after removing them and drying the seeds, samples were prepared for chemical analyses. All pea samples were ground prior to chemical analysis in the Cyclotec^TM^ laboratory mill (FOSS, Hillerød, Denmark) through a 0.5 mm sieve. All samples were stored in a fridge in sealed plastic cups until analysis.

### 3.2. Analytical Methods

Protein content was analyzed using the Kjeldahl method, as described in the AOAC 955.04 [42] procedure, using N × 6.25 as a conversion factor.

The amino acid composition of the protein was assessed according to the method of Mason et al. [43]. The protein profile was analyzed using ion-exchange chromatography with the AAA 400 amino acid analyzer (INGOS, Praha, Czech Republic). Samples were hydrolyzed with 6M HCl at 110 °C for 24 h after pre-oxidation of the sulfuric amino acids with performic acid. The results are expressed in g per 100 g of protein. The nutritional value of the protein was estimated based on the calculated chemical score (CS) and the essential amino acids index (EAAI) against the amino acid composition of the FAO protein reference standard (1965) [30].

Ash quantification was assessed gravimetrically, according to AOAC method 923.03 [42]. The determination of ash content was used as a measure of the total amount of minerals.

Total lipids content was determined gravimetrically by extraction with acid solvent consisting of 60:40:1 (*v*/*v*/*v*) chloroform, methanol and concentrated hydrochloric acid, as described by Marchello et al. [44].

Available starch was measured via the Megazyme procedure (Bray, Ireland), consistent with the AACC-approved method 76–16 [45].

Dietary fiber (DF) content was determined using the enzymatic chemical method in accordance with AACC 32–25 [45] and AOAC 994.13 [42] procedures as a sum of non-starch polysaccharides (NSP), raffinose family oligosaccharides (RFO), uronic acids (UA), resistant starch (RS) and lignin.

NSP content with its fractionation to soluble (S-NSP) and insoluble (I-NSP) fractions was determined using gas chromatography (GC) according to the method of Englyst and Cummings [46]. In this procedure, the NSP of each fraction is a sum of the seven individual monomers rhamnose, fucose, arabinose, xylose, mannose, galactose and glucose. After the enzymatic hydrolysis of starch, the samples were centrifuged and split into soluble (ethanol precipitates from supernatant) and insoluble (remaining pellet) fractions. Each of these fractions was hydrolyzed with 1 M sulfuric acid (100 °C, 2h) to monosaccharides and converted to volatile alditol acetates. The alditol acetates were separated on a capillary quartz column Rtx-225 (0.53 mm × 30 m) using the Clarus 600 gas chromatograph (Perkin Elmer, Waltham, MA, USA) equipped with an autosampler, splitter injection port and flame ionization detector. The carrier gas was He. Separation was performed at 225 °C, with injection and detection at 275 °C.

Raffinose family oligosaccharides (RFO) were analyzed as described by Lahuta [47]. In this procedure, oligosaccharides were extracted with 50% ethanol and then derivatized with a mixture of trimethyl imidazole and pirydyne (1:1, *v*/*v*). Derivatives were separated on a capillary quartz column Rtx-1 (0.25 mm × 15 m) using the Clarus 600 gas chromatograph (Perkin Elmer) equipped with an autosampler, splitter injection port and flame ionization detector. RFOs were calculated as a sum of raffinose, stachyose and verbascose.

Uronic acids (UA) were measured with the colorimetric method previously described by Scott [48], using 3.5-dimethylphenol, highly reactive to uronic acids derivatives. UA were calculated by measuring the difference between absorbance at 400 nm and 450 nm on a UV-VIS spectrometer (Thermo Fisher Scientific, Waltham, MA, USA). The solution of D-galacturonic acids was used as a standard.

Resistant starch was measured via the Megazyme procedure (Bray, Ireland), consistent with the AACC-approved method 32–40.01 [45].

Lignin and other insoluble residues were determined gravimetrically, as described by Theander and Westerlund [49], as a dry (105 °C, 16 h) residue of a sample previously digested with 72% sulfuric acid that had been incinerated (550 °C, 5 h). The percentage contents of lignin and associated polyphenols were calculated on the basis of the loss in weight by incinerating the dried insoluble material.

All chemical analyses were performed in duplicate and the mean value was accepted if the difference between duplicates was below 10%. All the obtained results were calculated on a dry weight basis (DW).

### 3.3. Statistical Analysis

To study the variability in the contents of nutrient and bioactive components within pea genotypes, a one-way fixed model of analysis of variance (ANOVA) and Tukey’s contrast analyses were performed. The significance level was set to α = 0.01 or α = 0.05 to evaluate significant differences. The Pearson correlation coefficients between analyzed traits were also calculated. Principal component analysis (PCA) was carried out to obtain an overview of differences in analyzed components between each pea genotype. All statistical analyses were performed using Statistica (data analysis software system), version 13.3.

## 4. Conclusions

Based on the obtained results, it can be stated that the analyzed genotypes constitute a valuable raw material, being a source of nutritional and health-promoting compounds, especially the protein and dietary fiber complex. The significantly higher contents of most of the tested components found in the analyzed breeding lines in comparison to the varieties indicate their high potential as a raw material for food production. The groups selected during the PCA analysis allowed for the identification of specific genotypes distinguished in terms of the contents of nutrients, bioactive substances, and all features combined. Among the breeding lines, three genotypes with the greatest potential in terms of the most important parameters were selected. The line WTD5006 was the most promising in terms of protein content in the group of tested genotypes, and it was characterized by a relatively high amount of dietary fiber. Genotypes with a very high protein content can be used in the production of protein supplements and granulates. The other two lines HRG-2 and HRG-3 were characterized by the highest contents of dietary fiber and high protein contents. Similar features were also observed for the Akord and Cysterski varieties. Such genotypes have the greatest potential for use as raw materials for the production of functional foods, which are rich sources of dietary fiber. The results clearly indicate the potential utility of the newest pea breeding lines in agri-food systems, and the high quality described confirms the potential for development in future breeding work.

## Figures and Tables

**Table 1:** Mean values, analysis of variance (ANOVA) F-statistics and HSD Tukey’s homogenous groups (for α = 0.05) of nutrient (g kg^−1^ DW) for analyzed pea genotypes.

	Name	Protein	Lipids	Ash	Starch
Breeding lines of pea	WTD 5001	242.2 ^ef^	32.2 ^bcd^	29.4 ^gh^	403.3 ^fghi^
	WTD 5002	230.0 ^gh^	31.4 ^bcde^	28.3 ^h^	431.5 ^cde^
	WTD 5003	244.4 ^ef^	31.2 ^bcdef^	31.0 ^de^	410.6 ^efghi^
	WTD 5004	216.5 ^ij^	32.8 ^b^	29.7 ^fg^	425.9 ^def^
	WTD 5005	211.0 ^j^	26.8 ^i^	26.4 ^i^	424.3 ^defg^
	WTD 5006	301.9 ^a^	36.9 ^a^	35.6 ^a^	268.9 ^j^
	WTD 5007	237.9 ^fg^	26.9 ^hi^	29.5 ^fg^	433.0 ^bcde^
	WTD 5008	230.8 ^gh^	29.3 ^efgh^	29.2 ^gh^	441.4 ^bcd^
	HR-G 1	260.6 ^bc^	27.3 ^hi^	32.7 ^c^	401.3 ^ghi^
	HR-G 2	259.4 ^bc^	28.7 ^ghi^	30.7 ^ef^	397.7 ^hi^
	HR-G 3	265.1 ^bc^	28.7 ^ghi^	29.1 ^gh^	392.6 ^i^
	HR-G 4	256.3 ^cd^	28.4 ^ghi^	32.6 ^c^	411.5 ^efghi^
	HR-G 5	237.7 ^fg^	30.2 ^cdefg^	29.5 ^fg^	411.5 ^efghi^
	HR-G 6	238.0 ^fg^	26.6 ^i^	29.0 ^gh^	425.4 ^def^
	HR-G 7	248.7 ^de^	32.3 ^bc^	29.8 ^fg^	418.2 ^defgh^
	HR-G 8	264.4 ^bc^	28.9 ^fghi^	32.1 ^cd^	402.2 ^fghi^
Pea varieties	Akord	226.1 ^hi^	32.6 ^bc^	35.4 ^a^	466.1 ^a^
	Cysterski	197.8 ^k^	29.8 ^defg^	34.0 ^b^	454.4 ^abc^
	Olimp	257.0 ^cd^	32.2 ^bc^	30.2 ^efg^	419.7 ^defgh^
	Starski	268.5 ^b^	30.6 ^bcdefg^	32.9 ^bc^	455.3 ^ab^
	Mean	244.7	30.2	30.9	414.7
	F statistics	170.9 **	37.8 **	130.0 **	92.0 **

Note: **—significant at *p* ≤ 0.01; means values with the same letters do not differ significantly.

**Table 2:** Mean values, analysis of variance (ANOVA) F-statistics and HSD Tukey’s homogenous groups (for α = 0.05) of the essential amino acids, their sum (EAA) and a sum of total amino acid (AA) (g/100 g of protein DW) and qualitative protein indices.

	Name	His	Ile	Leu	Lys	Met+Cys	Phe	Thr	Tyr	Val	Sum of EAA	Sum of AA	CS	EAAI
Breeding lines of pea	WTD 5001	2.8 ^a^	3.9 ^bcde^	7.0 ^abcde^	7.3 ^fg^	2.7 ^ab^	5.0 ^abc^	3.5 ^fg^	2.7 ^abc^	4.7 ^abcd^	36.8 ^fgh^	96.7 ^a^	47 met+cys	80
	WTD 5002	2.5 ^bc^	4.1 ^abcd^	7.2 ^ab^	7.8 ^bcdef^	2.5 ^bcd^	4.9 ^abc^	4.5 ^b^	2.5 ^c^	4.8 ^abc^	38.2 ^abcd^	93.6 ^bc^	43 met+cys	83
	WTD 5003	2.4 ^bc^	3.9 ^bcde^	7.2 ^ab^	7.1 ^g^	2.3 ^d^	4.8 ^bc^	3.3 ^g^	2.5 ^bc^	4.4 ^d^	35.5 ^i^	94.2 ^bc^	40 met+cys	76
	WTD 5004	2.5 ^bc^	3.7 ^e^	6.8 ^cde^	7.4 ^defg^	2.6 ^abc^	4.9 ^abc^	3.7 ^f^	2.6 ^bc^	4.5 ^cd^	36.2 ^hi^	94.5 ^abc^	45 met+cys	79
	WTD 5005	2.5 ^bc^	4.2 ^a^	7.3 ^a^	8.0 ^abc^	2.8 ^a^	5.0 ^abc^	3.7 ^f^	2.6 ^abc^	4.7 ^abc^	38.4 ^abcd^	94.7 ^abc^	49 met+cys	83
	WTD 5006	2.5 ^bc^	4.1 ^ab^	7.3 ^a^	8.3 ^ab^	2.7 ^ab^	5.1 ^bc^	3.5 ^fg^	2.6 ^abc^	4.7 ^bcd^	38.3 ^abcd^	95.1^abc^	47 met+cys	82
	WTD 5007	2.6^abc^	4.0^abcd^	7.1^abcde^	8.0^abc^	2.6^abc^	4.8^bc^	3.7^f^	2.5^bc^	4.7^abc^	37.3^efgh^	95.1^abc^	45 met+cys	81
	WTD 5008	2.5 ^bc^	4.1 ^abc^	7.2 ^a^	8.0 ^abc^	2.7 ^ab^	5.0 ^abc^	4.4 ^bc^	2.7 ^abc^	4.6 ^bcd^	38.8 ^abc^	93.9 ^bc^	48 met+cys	84
	HR-G 1	2.5 ^bc^	3.9 ^cde^	6.7 ^e^	7.7 ^cdefg^	2.7 ^ab^	4.7 ^c^	3.7 ^ef^	2.6 ^abc^	4.7 ^abcd^	36.6 ^ghi^	94.2 ^bc^	47 met+cys	80
	HR-G 2	2.5 ^bc^	3.8 ^cde^	7.1 ^abc^	7.7 ^bcdef^	2.4 ^cd^	4.8 ^bc^	3.7 ^f^	2.6 ^abc^	4.5 ^cd^	36.6 ^fghi^	95.3 ^ab^	41 met+cys	79
	HR-G 3	2.6 ^bc^	4.2 ^ab^	7.2 ^ab^	8.4 ^a^	2.8 ^a^	4.9 ^abc^	4.2 ^cd^	2.7 ^abc^	4.9 ^ab^	39.2 ^a^	94.4 ^abc^	49 met+cys	85
	HR-G 4	2.4 ^c^	4.1 ^abcd^	7.2 ^ab^	8.0 ^abc^	2.6 ^abc^	4.9 ^abc^	4.4 ^bc^	2.6 ^abc^	5.0 ^a^	38.9 ^ab^	93.8 ^bc^	46 met+cys	84
	HR-G 5	2.5 ^bc^	4.1 ^abc^	7.1 ^abc^	8.2 ^ab^	2.7 ^ab^	4.8 ^abc^	4.0 ^de^	2.7 ^ab^	4.8 ^abc^	38.5 ^abcd^	93.7 ^bc^	47 met+cys	83
	HR-G 6	2.4 ^c^	3.8 ^de^	6.7 ^de^	7.7 ^bcdef^	2.7 ^ab^	4.7 ^c^	3.7 ^ef^	2.6 ^abc^	4.6 ^bcd^	36.7 ^fghi^	94.1 ^bc^	48 met+cys	80
	HR-G 7	2.4 ^bc^	4.0 ^abcd^	7.3 ^a^	8.1 ^abc^	2.6 ^ab^	5.0 ^abc^	4.6 ^b^	2.5 ^bc^	4.9 ^ab^	39.1 ^a^	94.1 ^bc^	46 met+cys	84
	HR-G 8	2.5 ^bc^	4.1 ^abc^	6.9 ^bcde^	7.9 ^abcde^	2.7 ^ab^	4.8 ^bc^	5.2 ^a^	2.7 ^abc^	4.8 ^abc^	39.0 ^ab^	92.9 ^c^	48 met+cys	84
Pea varieties	Akord	2.7 ^ab^	4.0 ^abcd^	7.1 ^abcd^	7.4 ^efg^	2.7 ^ab^	5.0 ^abc^	3.5 ^fg^	2.8 ^a^	4.8 ^abc^	37.4 ^defg^	96.8 ^a^	48 met+cys	82
	Cysterski	2.6 ^abc^	4.1 ^abcd^	7.2 ^ab^	8.0 ^abc^	2.7 ^ab^	5.2 ^a^	3.8 ^ef^	2.7 ^abc^	4.7 ^abcd^	38.3 ^abcd^	95.1 ^abc^	48 met+cys	83
	Olimp	2.6 ^abc^	4.1 ^abcd^	7.2 ^ab^	8.1 ^abc^	2.7 ^ab^	4.7 ^bc^	3.7 ^ef^	2.5 ^c^	4.7 ^abcd^	37.6 ^cdefg^	93.9 ^abc^	47 met+cys	82
	Starski	2.5 ^bc^	4.0 ^abcd^	7.1 ^abc^	8.0 ^abcd^	2.7 ^ab^	4.9 ^abc^	3.7 ^ef^	2.7 ^abc^	4.8 ^abc^	37.9 ^bcdef^	94.3 ^bc^	48 met+cys	82
	Mean	2.5	4.0	7.1	7.9	2.6	4.9	3.9	2.6	4.7	37.8	94.6	46 met+cys	82
	F statistics	4.82 **	7.5 **	8.5 **	11.7 **	8.24 **	4.6 **	73.6 **	3.97 **	5.8 **	25.4 **	5.0 **	―	―

Note: **—significant at *p* ≤ 0.01; means values with the same letters do not differ significantly; His, histidine; Ile, isoleucine; Leu, leucine; Lys, lysine; Met, methionine; Cys, cysteine; Phe, phenylalanine; Thr, threonine; Tyr, tyrosine; Val, valine; EAA, essential amino acid; AA, sum of amino acids; CS, chemical score; EAAI, essential amino acid index.

**Table 3:** Mean values, analysis of variance (ANOVA) F-statistics and HSD Tukey’s homogenous groups (for α = 0.05) of the non-essential amino acids (NEAA) (g/100 g of protein DW).

	Name	Asp	Ser	Glu	Pro	Gly	Ala	Arg
Breeding lines of pea	WTD 5001	11.2 ^ef^	5.0 ^ab^	18.7 ^a^	4.1 ^abc^	4.4 ^ab^	4.3 ^fgh^	9.4 ^b^
	WTD 5002	12.9 ^a^	3.5 ^i^	16.0 ^gh^	3.4 ^cdef^	4.1 ^de^	4.8 ^abc^	8.2 ^def^
	WTD 5003	11.2 ^ef^	5.1 ^a^	17.6 ^bcde^	3.8 ^abcdef^	4.2 ^bcd^	4.1 ^h^	10.1 ^a^
	WTD 5004	11.1 ^f^	4.8 ^abc^	17.7 ^bcde^	4.5 ^a^	4.3 ^bcd^	4.2 ^gh^	9.3 ^b^
	WTD 5005	11.8 ^cde^	4.5 ^cde^	17.5 ^bcde^	3.8 ^bcdef^	4.2 ^bcd^	4.7 ^abcd^	7.3 ^ij^
	WTD 5006	12.0 ^bcd^	4.3 ^def^	17.7 ^bcd^	3.7 ^bcdef^	4.0 ^e^	4.5 ^defg^	8.1 ^defg^
	WTD 5007	11.5 ^def^	4.7 ^abcd^	18.0 ^abc^	4.2 ^ab^	4.2 ^bcd^	4.7 ^abcd^	7.9 ^efghij^
	WTD 5008	12.9 ^a^	3.7 ^hi^	16.1 ^gh^	3.2 ^f^	4.1 ^cde^	4.6 ^bcde^	8.0 ^efgh^
	HR-G 1	12.0 ^bcd^	4.6 ^cde^	18.1 ^ab^	3.6 ^bcdef^	4.4 ^ab^	5.0 ^a^	7.6 ^fghij^
	HR-G 2	12.3 ^abc^	4.6 ^cde^	18.1 ^ab^	4.0 ^abcd^	4.0 ^e^	4.5 ^cdef^	8.8 ^bcd^
	HR-G 3	11.5 ^def^	4.2 ^efg^	16.8 ^efg^	3.5 ^cdef^	4.3 ^ab^	4.8 ^abc^	7.5 ^ghij^
	HR-G 4	12.5 ^ab^	3.7 ^hi^	16.2 ^gh^	3.2 ^ef^	4.1 ^de^	4.7 ^abcd^	8.0 ^efghi^
	HR-G 5	11.4 ^def^	4.0 ^fgh^	16.9 ^defg^	3.6 ^bcdef^	4.2 ^bcd^	4.9 ^ab^	7.7 ^fghij^
	HR-G 6	12.0 ^bcd^	4.3 ^def^	17.9 ^abc^	3.8 ^bcdef^	4.4 ^ab^	5.0 ^a^	7.7 ^efghij^
	HR-G 7	12.7 ^a^	3.8 ^ghi^	16.4 ^fgh^	3.4 ^def^	4.1 ^cde^	4.6 ^bcde^	7.5 ^ghij^
	HR-G 8	12.3 ^abc^	3.4 ^i^	15.7 ^h^	3.7 ^bcdef^	4.3 ^bcd^	4.8 ^abc^	7.3 ^j^
Pea varieties	Akord	11.1 ^f^	5.1 ^a^	18.7 ^a^	4.1 ^abc^	4.5 ^a^	4.3 ^efgh^	8.9 ^bc^
	Cysterski	11.7 ^cdef^	4.6 ^bcd^	17.1 ^cdef^	3.6 ^bcdef^	4.2 ^bcd^	4.8 ^abcd^	8.0 ^efgh^
	Olimp	11.7 ^cdef^	4.4 ^cdef^	17.6 ^bcde^	3.7 ^bcdef^	4.2 ^bcd^	4.8 ^abcd^	8.3 ^cde^
	Starski	11.7 ^cdef^	4.6 ^cde^	17.2 ^bcdef^	3.9 ^abcde^	4.3 ^abc^	4.8 ^ab^	7.4 ^hij^
	Mean	11.9	4.3	17.3	3.7	4.2	4.6	8.1
	F statistics	21.7 **	43.98 **	30.1 **	7.49 **	14.7 **	22.3 **	47.9 **

Note: **—significant at *p* ≤ 0.01; mean values with the same letters do not differ significantly; Asp, aspartic acid; Ser, serine; Glu, glutamic acid; Pro, proline; Gly, glycine; Ala, alanine; Arg, arginine.

**Table 4:** Mean values, analysis of variance (ANOVA) F-statistics and HSD Tukey’s homogenous groups (for α = 0.05) of dietary fiber components (g kg^−1^ DW) for analyzed pea genotypes.

	Name	I-NSP	S-NSP	NSP	RFO	UA	RS	Lignin	DF
Breeding lines of pea	WTD 5001	112.1 ^gh^	10.3 ^abc^	122.4 ^gh^	88.3 ^a^	24.2 ^cde^	11.1 ^j^	6.7 ^efg^	252.7 ^hi^
	WTD 5002	109.2 ^gh^	9.6 ^bcde^	118.8 ^gh^	84.3 ^ab^	22.2 ^ef^	15.4 ^fgh^	8.3 ^def^	249.1 ^hij^
	WTD 5003	87.2 ^k^	8.8 ^efgh^	96.1 ^k^	87.2 ^a^	23.4 ^def^	10.3 ^j^	10.1 ^d^	227.1 ^lm^
	WTD 5004	92.9 ^jk^	9.5 ^cde^	102.4 ^jk^	87.4 ^a^	23.2 ^def^	18.5 ^e^	28.0 ^a^	259.5 ^gh^
	WTD 5005	104.5 ^hi^	10.7 ^ab^	115.2 ^hi^	83.1 ^ab^	23.1 ^def^	15.1 ^gh^	30.2 ^a^	266.7 ^fg^
	WTD 5006	128.0 ^f^	9.3 ^cdef^	133.3 ^f^	79.7 ^bc^	30.7 ^b^	41.5 ^a^	4.7 ^ghi^	293.9 ^cd^
	WTD 5007	97.7 ^ij^	9.4 ^cdef^	107.0 ^ij^	74.2 ^cd^	21.5 ^fg^	12.2 ^ij^	2.7 ^i^	217.6 ^m^
	WTD 5008	95.5 ^jk^	9.0 ^defgh^	104.5 ^jk^	82.0 ^ab^	24.3 ^cde^	17.6 ^ef^	4.0 ^hi^	232.3 ^kl^
	HR-G 1	160.6 ^d^	7.5 ^i^	168.0 ^d^	80.1 ^bc^	25.0 ^cd^	14.2 ^hi^	9.4 ^d^	296.7 ^c^
	HR-G 2	218.2 ^a^	9.8 ^abcde^	228.0 ^a^	70.6 ^d^	24.3 ^cde^	17.0 ^efg^	22.5 ^b^	362.4 ^a^
	HR-G 3	197.7 ^b^	9.9 ^abcde^	207.6 ^b^	81.8 ^ab^	24.9 ^cd^	16.1 ^fgh^	20.4 ^c^	351.0 ^a^
	HR-G 4	172.8 ^c^	8.1 ^hi^	180.9 ^c^	82.0 ^ab^	24.9 ^cd^	20.8 ^cd^	19.8 ^c^	328.4 ^b^
	HR-G 5	108.0 ^gh^	9.0 ^defgh^	117.0 ^gh^	70.5 ^d^	26.3 ^c^	14.5 ^h^	8.5 ^de^	236.9 ^jkl^
	HR-G 6	111.7 ^gh^	9.2 ^defgh^	120.9 ^gh^	73.5 ^cd^	26.0 ^c^	10.9 ^j^	4.5 ^ghi^	235.8 ^kl^
	HR-G 7	112.5 ^gh^	10.0 ^abcd^	122.4 ^gh^	69.2 ^d^	30.0 ^b^	11.5 ^j^	9.3 ^d^	242.5 ^ijk^
	HR-G 8	1380 ^e^	9.2 ^cdefg^	147.2 ^e^	82.3 ^ab^	33.9 ^a^	10.5 ^j^	9.5 ^d^	283.4 ^de^
Pea varieties	Akord	136.2 ^ef^	10.9 ^a^	147.1 ^e^	86.7 ^a^	19.7 ^gh^	30.7 ^b^	4.2 ^hi^	288.4 ^cd^
	Cysterski	144.0 ^e^	9.6 ^bcde^	153.6 ^e^	75.0 ^cd^	23.0 ^def^	19.1 ^de^	3.7 ^i^	274.4 ^ef^
	Olimp	113.9 ^g^	8.3 ^fghi^	122.2 ^gh^	69.8 ^d^	18.4 ^h^	14.6 ^h^	4.1 ^hi^	229.0 ^lm^
	Starski	116.7 ^g^	8.1 ^ghi^	124.8 ^g^	82.4 ^ab^	21.3 ^fg^	22.2 ^c^	6.1 ^fgh^	256.8 ^gh^
	Mean	127.9	9.3	137.2	79.5	24.5	17.2	10.8	269.2
	F statistics	517.9 **	19.77 **	570.2 **	30.52 **	91.71 **	370.64 **	436.41 **	335.9 **

Note: **—significant at *p* ≤ 0.01; mean values with the same letters do not differ significantly; I-NSP, insoluble fraction of non-starch polysaccharides; S-NSP, soluble fraction of non-starch polysaccharides; NSP, non-starch polysaccharides, RFO, raffinose family oligosaccharides; UA, uronic acids; RS, resistant starch; DF, dietary fiber.

## Data Availability

The data presented in this study are available on request from the corresponding author due to privacy.
